# Management of Status Epilepticus in Children

**DOI:** 10.3390/jcm5040047

**Published:** 2016-04-13

**Authors:** Douglas M. Smith, Emily L. McGinnis, Diana J. Walleigh, Nicholas S. Abend

**Affiliations:** Departments of Neurology and Pediatrics, The Perelman School of Medicine at the University of Pennsylvania, The Children’s Hospital of Philadelphia, Philadelphia, PA 19104, USA; SmithD13@email.chop.edu (D.M.S.); RobbinsE@email.chop.edu (E.L.M.); WalleighD@email.chop.edu (D.J.W.)

**Keywords:** status epilepticus, pediatric, seizure

## Abstract

Status epilepticus is a common pediatric neurological emergency. Management includes prompt administration of appropriately selected anti-seizure medications, identification and treatment of seizure precipitant(s), as well as identification and management of associated systemic complications. This review discusses the definitions, classification, epidemiology and management of status epilepticus and refractory status epilepticus in children.

## 1. Introduction

Status epilepticus is characterized by prolonged or recurrent seizures without a return to baseline. It is a common pediatric neurological emergency with an estimated incidence of 18–23 per 100,000 children per year and a mortality of 2%–7% [1]. Management includes prompt administration of appropriately selected anti-seizure medications, identification and management of any seizure precipitant(s), as well as identification and management of associated systemic complications.

## 2. Definitions and Classification

Historically, status epilepticus has been conceptually defined as “a condition characterized by an epileptic seizure that is sufficiently prolonged or repeated at sufficiently brief intervals so as to produce an unvarying and enduring epileptic condition” [2]. This conceptual definition was pragmatically defined by the International League Against Epilepsy as 30 min of continuous seizure activity, or a series of epileptic seizures during which function is not regained between ictal events in a longer than 30 min period [3]. This definition was based on animal studies demonstrating acute changes in hippocampal neurons after 30–90 min of chemically-induced seizure activity [4] and that prolonged seizures led to brain injury in paralyzed and mechanically ventilated baboons [5]. However, this definition was problematic for clinicians who aimed to aggressively treat status epilepticus earlier before any neuronal injury might occur. This clinical goal was driven by studies demonstrating that longer seizures predict a longer total duration of status epilepticus and poorer prognosis [6]. Furthermore, it was recognized that these risks of mortality and pathophysiologic changes occurred at varying seizure durations depending on seizure type and seizure etiology. Thus, more clinically focused definitions have been developed. The 2015 International League Against Epilepsy defines status epilepticus as “a condition resulting either from the failure of the mechanisms responsible for seizure termination or from the initiation of mechanisms which lead to abnormally prolonged seizures (after time point t_1_). It is a condition that can have long-term consequences (after time point t_2_), including neuronal death, neuronal injury, and alteration of neuronal networks, depending on the type and duration of seizures”. These time points were variably defined depending on whether the seizure was generalized tonic-clonic status epilepticus, focal status epilepticus with impaired consciousness, or absence status epilepticus (Table 1) [7]. Thus, the definition recognizes the variable urgency in treating status epilepticus depending on the type of seizure, and it distinguishes between status epilepticus with motor features and without prominent motor features (*i.e.*, non-convulsive status epilepticus). However, it does not establish a separate treatment approach for non-convulsive status epilepticus. A similar management approach was proposed by the 2012 Neurocritical Care Society’s Guideline on the Evaluation and Management of Status Epilepticus. This guideline defines status epilepticus as five minutes of continuous clinical or electrographic seizure activity, and it establishes the goal of achieving definitive control of status epilepticus within 60 min of onset [8]. The 2016 American Epilepsy Society’s Guideline for Status Epilepticus Management follows the five minute definition without subdividing based on seizure type [9].

Terminology used to describe different seizure stages/phases reflects the complex and nuanced definition of status epilepticus. The first five minutes of a seizure have been termed “prodromal” or “incipient status epilepticus” [10]. Continued seizure activity can be further subdivided into early status epilepticus (5–30 min), established status epilepticus (>30 min), and refractory status epilepticus (ongoing status epilepticus despite administration of 2–3 appropriately dosed anti-seizure medications). Per the Neurocritical Care Society’s guideline, medical interventions to stabilize the patient and identify any underlying precipitant are categorized as “immediate”, which roughly corresponds to incipient status epilepticus. Anti-seizure medications are classified as “emergent”, “urgent”, or “refractory”. Emergent interventions correspond with the temporal definitions of early status epilepticus, and urgent interventions with established status epilepticus. These semantic changes forsake classifying the order of medication in favor of emphasizing the importance of rapid sequential medication administration. The American Epilepsy Society’s guideline provides a hybrid system in which the medication order is defined but the timeframes for each phase are shorter than provided by some prior systems. It suggests management be considered as an “initial therapy phase” (5–20 min), a “second therapy phase” (20–40 min), and a “third therapy phase” (40–60 min) [9].

Less important than the exact time defined as the point of transition from seizure to status epilepticus is the emphasis placed on prompt and aggressive management. As recognized by the concept of t_1_ in the International League Against Epilepsy’s consensus statement, after a certain period of time, a threshold is crossed where a seizure becomes unlikely to self-terminate. During this time, pathophysiologic changes occur that promote pharmacoresistance, reducing the likelihood that status epilepticus will respond to initial anti-seizure medications. In children with new-onset seizures, after continuously seizing for 5–10 min, a seizure becomes unlikely to stop without pharmacologic intervention [11]. Several studies have described associations between status epilepticus management delays and more prolonged seizures [12] and lower anti-seizure medication responsiveness [13,14,15,16]. These data underscore the need to initiate management for seizures lasting more than about 5–10 min.

Despite the need for prompt intervention to identify any underlying etiology and terminate seizures called for by recent guidelines [8,9], substantial variability in care and delays in care are common in both the pre-hospital and in-hospital settings [17]. Studies of status epilepticus management in children in emergency departments have described that laboratory parameters were often not checked and some results were only available after long delays [18], benzodiazepine dosing was outside usual dosing guidelines in 23% of children with status epilepticus [18], the median time to administer a second-line anti-seizure medication to a seizing child was 24 min [19], and substantial delays in anti-seizure medication administration are common in children with refractory status epilepticus [17]. To expedite therapeutic decisions, a consensus document recommended that all units have a written management pathway with a clear structured time frame [20]. Several example pathways have been published [8,9,10,21,22], but these may need to be adapted based on local resources and practices. Additionally, many of the pathways provide management recommendations intended for generalized convulsive status epilepticus and focal status epilepticus with impaired consciousness while management of other forms of status epilepticus, such as focal status epilepticus with preserved consciousness and non-convulsive status in patients with acute encephalopathy, may require more individualized approaches.

## 3. Epidemiology, Morbidity, and Mortality

Current estimates of the incidence of status epilepticus in children vary by age. The incidence is highest in the neonatal period and declines until approximately five years of age [1,23,24,25,26]. Estimates in the neonatal to first year of life are approximately 135–150 incidents per 100,000 people [24,25], with higher incidence in vulnerable populations with acute or chronic neurologic conditions. This population also has a much higher incidence of acute symptomatic causes of status epilepticus [1,23]. The incidence of status epilepticus is relatively low between the ages of 5 and 40 years [24]. Across the pediatric lifespan, the most recent comprehensive prospective estimate of the incidence of status epilepticus is between 17 and 23 per 100,000 children [1]. A more recent population-based study estimates the incidence between 5 and 15 per 100,000 people [27]. Epidemiologic features unique to the pediatric population include a relatively higher rate of recurrence of status epilepticus [24], more frequent provoking infectious [24] or remote symptomatic causes [24,28], and more likely occurrence in children without a diagnosis of epilepsy [29]. In over 75% of cases status epilepticus may be the first seizure of life [23], and children presenting with status epilepticus as their first seizure only have a 30% risk of a later diagnosis of epilepsy [28].

Despite the higher incidence of status epilepticus in children than adults, the overall mortality of status epilepticus is lower in children than in adults [27]. In some studies, age is the only factor in multivariate analysis to predict a better outcome [26]. While mortality was higher in early studies [23], recent studies with prospective measurement in children approximate mortality at 3% [1]. Mortality and morbidity directly from status epilepticus is difficult to distinguish from the underlying cause of the seizure.

## 4. Medical Management and Stabilization

Status epilepticus is a medical as well as neurologic emergency. Medical stabilization focuses on providing support of airway, breathing, and circulatory functions while identifying medical complications and seizure precipitants. Medical management should proceed with subsequent testing once stabilization of airway, breathing, and circulation occurs. This may require intubation with mechanical ventilation to support pulmonary function and vasopressors and fluid resuscitation to support circulation. As hyperthermia [30] and hyperglycemia [31] are associated with unfavorable outcomes in some types of neurologic injury which may cause status epilepticus, close attention to these parameters is recommended. Neurologically, seizure management involves providing definitive treatment of both clinical and electrographic seizure activity while simultaneously performing an investigation aimed at identifying the cause of the seizure. The Neurocritical Care Society’s guideline provides a timed treatment outline for this critical time period [8]. Steps to be completed in the “immediate” (initial five minute) time frame include non-invasive airway positioning, assessment of adequacy of ventilation and perfusion by checking vital signs, establishing a means of peripheral intravenous access, checking a finger-stick glucose, and checking a set of baseline triage labs. Once intravenous access is established, an “emergent” anti-seizure medication (*i.e.*, a benzodiazepine) is administered. If intravenous access cannot be rapidly established, other routes of benzodiazepine administration should be used including intramuscular, rectal, buccal, and nasal.

## 5. Diagnostics

Multiple studies have investigated pediatric status epilepticus etiologies, and febrile status epilepticus is the most common diagnosis [32,33,34]. Precipitant categories include acute symptomatic, remote symptomatic, acute-on-remote symptomatic, cryptogenic, and idiopathic. Acute symptomatic and acute-on-remote symptomatic causes, which comprise 17% to 26% of cases of pediatric status epilepticus, respectively [23,32,33,34], should be evaluated urgently, as addressing these precipitants may simultaneously treat seizures. The American Academy of Neurology practice parameter addressing the diagnostic assessment of a child with convulsive status epilepticus reported that abnormal results among children who underwent testing included low anti-seizure medication levels (32%), neuroimaging abnormalities (8%), electrolytes (6%), inborn errors of metabolism (4%), ingestion (4%), central nervous system infections (3%), and positive blood cultures (3%) [35]. To identify these precipitants, the Neurocritical Care Society’s guideline recommends a finger-stick glucose in the initial two minutes as well as a serum glucose, complete blood count, basic metabolic panel, blood gas, calcium, magnesium, and anti-seizure medication levels drawn in the initial five minutes [8]. Rapidly correctable causes of status epilepticus should be identified and treated as quickly as possible, including hypoglycemia, hypocalcemia, hyponatremia, and hypomagnesemia. Some patients may require additional diagnostic testing including lumbar puncture, neuroimaging, and other blood work (liver function tests, coagulation panel, serum or urine drug screen, inborn errors of metabolism screen), which are recommended to be performed in the initial hour. Testing for rarer causes of status epilepticus, including specific antibodies or PCR for viral encephalitides, autoantibody testing, or metabolic testing, may be considered in some patients [36].

Consideration should also be given to performing continuous electroencephalogram (EEG) monitoring. The Neurocritical Care Society’s guideline stipulates that EEG monitoring should be initiated 15–60 min after seizure onset to evaluate for non-convulsive status epilepticus for patients who are not returning to baseline within 10 min of convulsive seizure cessation or within 60 min for patients in whom ongoing seizures are suspected. Further, the guideline recommends 48 h of EEG monitoring in critically ill encephalopathic patients to identify non-convulsive status epilepticus, including patients who are comatose, have intracerebral hemorrhage of any type, with epileptiform discharges on a routine EEG, or who have altered mental status with suspected non-convulsive seizures [8]. A consensus statement from the American Clinical Neurophysiology Society regarding EEG monitoring in critically ill adults and children recommends written plans for EEG monitoring (including indications, urgency, and duration), obtaining time-locked video, and EEG monitoring review at least twice per day. The consensus statement provides EEG monitoring indications which include: (1) persistently altered mental status after convulsive status epilepticus or seizures terminate; (2) acute supratentorial brain injury (including intraparenchymal hemorrhage, moderate-severe traumatic brain injury, central nervous system infections, recent neurosurgical procedures, brain tumors, acute ischemic stroke, hypoxic-ischemic encephalopathy, sepsis associated encephalopathy, extracorporeal membrane oxygenation) with altered mental status; (3) fluctuating or unexplained alteration in mental status; (4) routine EEG with periodic discharges; (5) pharmacologic paralysis and risk for seizures; or (6) paroxysmal events suspected to be seizures. The consensus statement recommends EEG monitoring for 24 h, the entire duration of pharmacologic coma induction for seizure management, and for 24 h after pharmacologic coma drugs weaned [37,38]. Additionally, urgent EEG may be indicated when non-epileptic seizures are suspected (e.g., psychogenic status epilepticus), as the appropriate diagnosis may avoid further administration of unnecessary anti-seizure medications.

As the principal management goal is cessation of both clinical and electrographic seizure activity [8], continuous EEG monitoring should be considered in all children following convulsive status epilepticus with persisting altered mental status [8,37]. In a multi-center cohort of critically ill children, 33% of 98 children who presented with convulsive status epilepticus had electrographic seizures identified [39]. The overall seizure burden was high, with electrographic status epilepticus occurring in 47% of patients with seizures identified with EEG monitoring. Further, 34% of children with seizures had exclusively EEG-only seizures which would not have been identified without EEG monitoring [40]. Additionally, EEG monitoring performed in encephalopathic critically ill children with or without prior clinically evident seizures often impacts clinical management [41] through identification of non-convulsive seizures and non-convulsive status epilepticus persisting after control of convulsive status epilepticus or clinically evident seizures [42]. Observational studies have reported that in multivariable analyses aiming to account for encephalopathy etiology and severity, high electrographic seizure exposures in critically ill children are associated with worse outcomes [42,43,44,45,46]. As a result of these data, continuous EEG monitoring is being used with increasing frequency in critically ill encephalopathic children to identify non-convulsive seizures and status epilepticus, including after termination of convulsive status epilepticus [47]. Further study is needed to better target limited EEG monitoring resources to the patients at highest risk for non-convulsive seizures and to determine whether efforts to identify and manage these electrographic seizures improve patient outcomes.

While there is agreement that patients with new-onset status epilepticus require neuroimaging [48], there is less of a consensus regarding the timing of imaging. The American Academy of Neurology’s Practice Parameter states that neuroimaging should be performed after the child is stabilized and the status epilepticus has been controlled [35]. The Neurocritical Care Society’s guideline considers imaging to be “urgent” and performed within the first 60 min of status epilepticus onset [8]. Multiple studies have explored the yield of imaging in new-onset epilepsy. As noted in the International League Against Epilepsy subcommittee recommendations, nearly 50% of individual imaging studies in children with localization-related new-onset seizure(s) were reported to be abnormal; 15%–20% of imaging studies provided useful information regarding seizure etiology and/or seizure focus, and 2%–4% provided information that potentially altered immediate medical management [49]. Fewer studies have explored the yield of imaging specifically for status epilepticus. One study determined that 20% of brain CTs and 58% of brain MRIs were abnormal in pediatric status epilepticus, with neuroimaging altering acute management in 24% of patients [33]. The necessity and timing of neuroimaging is at the discretion of the treating clinician. In cases where the cause of status epilepticus is clearly established, it may be appropriate to forego immediate neuroimaging. In other cases, such as in trauma or oncology patients or when the status epilepticus etiology is unknown, neuroimaging should be considered more urgently. In some cases, clinicians may need to decide between neuroimaging and EEG monitoring, although introduction of CT and MRI compatible electrodes has allowed both to be performed more easily. CT is typically performed first as it is more widely available than MRI, is less expensive, and is less likely to require sedation for younger children. If no etiology is revealed with CT, MRI should still be considered. In one series, 12% of children with status epilepticus and a normal CT had abnormalities on MRI [33].

Central nervous system infections are a common cause of pediatric status epilepticus, accounting for 1%–12% of subjects in a series from developed countries [50]. Infections include acute bacterial meningitis; acute viral meningitis or encephalitis; and subacute viral, bacterial, fungal, or parasitic meningitis. The most common infectious etiologies vary by age, acuity, whether or not the patient is immunocompromised, use of steroids or other immunosuppression, recent travel, animal exposures, season, and associated signs and symptoms. Initial evaluation in all status epilepticus patients without an obvious non-infectious etiology includes lumbar puncture with cell counts, gram stain, culture, protein, glucose, and herpes simplex virus PCR in appropriate patients. Guidelines from the Infectious Disease Society of America for adults recommend obtaining screening head imaging prior to lumbar puncture in patients who are immunocompromised, have a known space-occupying lesion or shunt, papilledema, or a focal neurological deficit [51]. Infectious etiologies have been reviewed recently [52].

Status epilepticus secondary to autoimmune etiologies is less common but still well-described. In a recent prospective cohort study in adults, autoimmune and paraneoplastic etiologies accounted for 2.5% of cases of status epilepticus [53]. Autoimmune encephalitides may be classified as either limbic or diffuse, and either paraneoplastic or non-paraneoplastic [54]. Presentation is variable depending on the category and on the specific autoimmune condition. Clinicians should suspect an autoimmune encephalitis in the presence of an otherwise unexplained cerebrospinal fluid pleocytosis or elevated protein, evidence of inflammation of limbic structures on MRI, recent-onset systemic symptoms concerning for malignancy, or when status epilepticus occurs as part of a subacute neurologic degenerative disorder. Further evaluation should include cerebrospinal fluid oligoclonal bands, IgG synthesis rate, and an IgG index to assess for intrathecal synthesis of antibodies. Anti-Hu antibodies are most frequently implicated overall in seizures, status epilepticus, and epilepsia partialis continua [54], while anti-NMDA receptor antibody encephalitis is the most well-described autoantibody-associated disease in the pediatric population [55,56]. Specific clinical features associated with autoantibody-associated conditions include concurrent recent-onset psychiatric disease, movement disorder, or hemiparesis in anti-NMDA receptor encephalitis; hyponatremia and facio-brachial-dystonic seizures in anti-LGI1 encephalitis; and stiff-person syndrome and cerebellar signs with GAD-1 autoantibodies [54]. Targeted antibody testing for anti-NMDAR antibodies and paraneoplastic autoantibody screens are commercially available. In general, testing cerebrospinal fluid for autoantibodies is considered more sensitive and specific than serum. Autoantigens associated with seizures and status epilepticus have been reviewed recently [54].

Genetic testing is not described in the Neurocritical Care Society’s guideline and typically does not play a role in the acute management of status epilepticus. However, multiple genetic causes of epilepsy are associated with recurrent status epilepticus and some may present with new-onset status epilepticus without specific diagnostic features on imaging or EEG. Dravet syndrome is a genetic form of epilepsy associated with recurrent status epilepticus, particularly after vaccinations, during febrile illnesses, and upon exposure to warm temperatures [57]. In up to 70% of patients, Dravet syndrome is associated with mutations in the gene SCN1A, which encodes a voltage-gated sodium channel. Children with Dravet syndrome are more likely to present with status epilepticus before the age of 18 months, and they are more likely to have recurrent status epilepticus [58]. Sodium channel blocking anti-seizure medications may worsen epilepsy in Dravet syndrome, but phenytoin has been reported as effective in managing status epilepticus in patients with Dravet syndrome [59]. Mutations in POLG1, the gene encoding a subunit of the enzyme polymerase γ, which is involved in mitochondrial DNA replication, are associated with Alpers’ disease [60]. Alpers’ disease involves infantile-onset epilepsy (particularly epilepsia partialis continua), ataxia, developmental regression, cortical blindness, and a slowly progressive liver failure. Previously normal children presenting with new-onset status epilepticus have been described [60], and it should be considered in new-onset epilepsia partialis continua if there is concurrent liver failure and if occipital rhythmic high-amplitude delta with superimposed polyspikes (RHADS) are seen on EEG [60]. Acute liver failure is described in several children shortly after valproic acid initiation [61] so valproic acid should be avoided in children with Alpers’ disease or patients suspected of having a POLG1 mutation. There are many other single gene and contiguous gene disorders associated with epilepsy and status epilepticus. Targeted Next Generation sequencing of known epilepsy-related genes is readily commercially available and is high-yield for severe epilepsy phenotypes [62]. While the turnaround time on genetic testing precludes utility in the early management of status epilepticus, clinicians should consider epilepsy gene panels or genome-wide analysis as part of the systematic, comprehensive evaluation of the child with epilepsy or status epilepticus when other etiologies are not identified.

## 6. Management of Status Epilepticus

Administration of appropriate anti-seizure medications should occur as the patient is medically stabilized and diagnostic studies are performed. Table 2 provides a summary of recommended medications and doses. The Neurocritical Care Society’s guideline states that benzodiazepines remain the “emergent initial therapy” of choice based both on available evidence and expert consensus. When possible, intravenous benzodiazepine administration is preferred. However, formulations exist for buccal, intranasal, intramuscular, and rectal administration, and these should be administered if intravenous access cannot be rapidly established. The American Epilepsy Society’s guideline concludes that intravenous lorazepam and diazepam are efficacious at stopping seizures lasting at least five min (level A evidence) and that rectal diazepam, intramuscular midazolam, intranasal midazolam, and buccal midazolam are probably effective at terminating seizures lasting at least five minutes (level B evidence) [9]. It concludes that there are three equivalent first-line options including intravenous lorazepam (0.1 mg/kg/dose; repeat once if needed), intravenous diazepam (0.15–0.2 mg/kg/dose; repeat once if needed) and intramuscular midazolam (10 mg for >40 kg; 5 mg for 13–40 kg; single dose) (level A evidence) [9]. A recent large randomized clinical trial compared lorazepam and diazepam in children with convulsive status epilepticus. Cessation of status epilepticus for 10 min without recurrence in 30 min occurred in 72% of the diazepam group and 73% of the lorazepam group. Assisted ventilation was required in 16% of the diazepam group and 18% of the lorazepam group. The only difference in secondary outcomes was that patients in the lorazepam group were more likely to be sedated than those in the diazepam group (67% *vs.* 50%). The study concluded that the data did not support preferential use of lorazepam over diazepam [63].

Administration of benzodiazepines may result in respiratory depression and hypotension, so continued monitoring and stabilization should occur. The American Epilepsy Society’s guideline concludes that respiratory depression was the most common adverse event associated with anti-seizure medication treatment (level A evidence) and that there was no difference in respiratory depression between midazolam, lorazepam, and diazepam by any administration route (level B evidence) [9]. A large randomized clinical trial of pediatric convulsive status epilepticus reported that assisted ventilation was required in 16% of the diazepam group and 18% of the lorazepam group [63]. If the seizure does not terminate 5–10 min following initial benzodiazepine administration, then a second benzodiazepine dose should be administered. However, care should also be taken to assess whether pre-hospital administration of a benzodiazepine occurred, as excess benzodiazepine dosing increases the risk of respiratory suppression [64].

Second-line medications are referred to as “urgent” medications by the Neurocritical Care Society guideline [8] and “second therapy phase” by the American Epilepsy Society guideline [9]. If status epilepticus is already established, then benzodiazepines alone will obtain seizure control in less than half of children [19]. Thus, the Neurocritical Care Society’s guideline recommends that following benzodiazepine administration, another “urgent control medication” should be administered [8]. Only limited data are available regarding the comparative effectiveness of second-line “urgent” anti-seizure medications [65]. The American Epilepsy Society’s guideline concludes that there was insufficient evidence to evaluate phenytoin or levetiracetam as second-line therapy (level U evidence) but that intravenous valproic acid has similar efficacy but better tolerability than intravenous phenobarbital (level B evidence) [9]. The NIH funded Established Status Epilepticus Treatment Trial (ESETT) will compare phenytoin, valproate, and levetiracetam for convulsive status epilepticus in children and adults, providing important data regarding these “urgent” medications [66].

In surveys of pediatric emergency providers [67] and neurologists [68], phenytoin or fosphenytoin remain the most-used anti-seizure medications if status epilepticus persists after administration of benzodiazepines. However, this historical role is based on few data that these medications are more effective than other options such as levetiracetam, phenobarbital, or valproate. For example, a recent meta-analysis of drugs administered for benzodiazepine refractory convulsive status epilepticus found phenytoin had lower efficacy (50%) than levetiracetam (69%), phenobarbital (74%), and valproate (76%) [65]. Phenytoin and fosphenytoin, a prodrug rapidly converted to phenytoin, inhibit voltage-gated sodium channels and thereby reduce excitability [69]. It is effective for focal epilepsy, but it may be ineffective and worsen seizures in some patients with generalized epilepsy. The Neurocritical Care Society’s guideline classifies phenytoin and fosphenytoin as appropriate emergent, urgent, or refractory status epilepticus treatments with an intravenous loading dose at 20 mg/kg (or for fosphenytoin, 20 phenytoin equivalents/kg) [8]. The American Epilepsy Society’s guideline concludes that there were insufficient data to compare phenytoin and fosphenytoin for efficacy (level U evidence) but that fosphenytoin is better tolerated than phenytoin (level B evidence), and that fosphenytoin is therefore preferred based on tolerability (although phenytoin is acceptable) (level B evidence) [9]. Cardiac arrhythmias and a local severe skin reaction may be seen secondary to phenytoin, and they are less common with fosphenytoin. Phenytoin is a strong hepatic enzyme inducer and may lower other drug levels.

Valproic acid is a broad-spectrum anti-seizure medication which modulates sodium channels, calcium channels, and the metabolism of GABA [69]. It is effective in the treatment of both generalized and focal epilepsy and may be more effective in treating status epilepticus in children than in adults [70]. In two recent meta-analyses, valproic acid was found to have the highest relative efficacy among typical second-line anti-seizure medications [65,70]. For example, one meta-analysis of drugs administered for benzodiazepine refractory convulsive status epilepticus found that valproate had higher efficacy (78%) than phenytoin, phenobarbital, and levetiracetam [65]. Valproate may be administered rapidly intravenously and is classified as an appropriate “emergent”, “urgent”, or “refractory” status epilepticus medication by the Neurocritical Care Society’s guideline at a typical intravenous loading dose of 20–40 mg/kg [8]. The American Epilepsy Society’s guideline recommends valproic acid dosing of 40 mg/kg [9]. Adverse events are infrequent with intravenous administration but include hypotension, thrombocytopenia, pancytopenia, platelet dysfunction, hypersensitivity reactions, pancreatitis and hyperammonemia. There is also a Federal Drug Administration black box warning for hepatotoxicity, which is highest in children who are under the age of two years, receiving anticonvulsant polypharmacy, and/or suspected of having mitochondrial or metabolic disorders. Valproate is a strong hepatic enzyme inhibitor and may raise other drug levels.

Phenobarbital is a positive allosteric modulator of GABA_A_ receptors and is indicated by the Neurocritical Care Society’s guideline as an “emergent”, “urgent”, or “refractory” status epilepticus medication [8]. The typical intravenous loading dose is 20 mg/kg, with an additional 5–10 mg/kg if needed. A recent meta-analysis of drugs administered for benzodiazepine refractory convulsive status epilepticus found that phenobarbital was efficacious in 74% of patients [65]. Phenobarbital is sedating and may result in respiratory depression or hypotension; if no artificial airway is present, clinicians should be prepared to intubate prior to initiating infusion. Phenobarbital is a strong liver enzyme inducer and may lower other drug levels. It has a half-life of up to 72 h and may be longer in patients with hepatic dysfunction.

Levetiracetam is an additional anti-seizure medication option to be considered for “urgent” therapy [8]. The mechanism of action is incompletely understood, but is noted to bind to a presynaptic vesicle glycoprotein and reduce neurotransmitter release [71]. Previously considered only for super-refractory status epilepticus, it has been used more often early for status epilepticus due to its ease of dosing and lack of drug interactions. Limited retrospective and observational studies have reported seizure cessation in some patients with levetiracetam [72,73,74,75,76] at intravenous loading doses of 20–60 mg/kg, with a loading dose of 60 mg/kg recommended by the American Epilepsy Society’s guideline [9]. A meta-analysis of drugs administered for benzodiazepine refractory convulsive status epilepticus found levetiracetam was efficacious in 69% of subjects [65]. Aggression has been described as an adverse event. Dose adjustment is needed in children with renal dysfunction.

## 7. Management of Refractory Status Epilepticus

Refractory status epilepticus is characterized by seizures that persist despite treatment with adequate doses of initial anti-seizure medications. Definitions for refractory status epilepticus have varied in seizure durations (no time criteria, 30 min, one hour, or two hours) and/or lack of response to different numbers (two or three) of anti-seizure medications. The Neurocritical Care Society’s guideline indicates that refractory status epilepticus is diagnosed when clinical or electrographic seizures persist after adequate doses of an initial benzodiazepine followed by a second appropriate anti-seizure medication [8]. In contrast to prior definitions of refractory status epilepticus, there is no specific time that must elapse to define refractory status epilepticus, thereby emphasizing the importance of rapid sequential treatment. Refractory status epilepticus occurs in about 10%–40% of children with status epilepticus [14,15,77]. Studies in children have indicated that status epilepticus lasted more than one hour in 26%–45% of patients [28,78], longer than two hours in 17%–25% of patients [78,79], and longer than four hours in 10% of patients [78].

In some patients, refractory status epilepticus may last many weeks or months despite treatment with multiple medications, which has been referred to as malignant refractory status epilepticus [80] or super-refractory status epilepticus [81,82]. This condition has also been referred to as de-novo cryptogenic refractory multi-focal status epilepticus [83], new-onset refractory status epilepticus (NORSE) [84,85,86], and febrile infection-related epilepsy syndrome (FIRES) [87,88,89]. Some of these entities in which refractory status epilepticus occurs in a previously healthy person with no identified cause except a recent infection may represent overlapping terms describing similar or identical entities [90]. This extremely refractory form of status epilepticus has been associated with infectious or inflammatory etiologies, younger age, previous good health, and high morbidity and mortality [80,83,84].

The management of refractory status epilepticus has been reviewed previously in children [10,21,91,92,93,94]. While there is variability in suggested pathways and reported management decisions [95], all pathways either administer additional anti-seizure medications, such as phenytoin/fosphenytoin, phenobarbital, valproate sodium, or levetiracetam, or proceed to pharmacologic coma induction with intravenous or inhaled medications. The Neurocritical Care Society’s guideline indicates that appropriate options for refractory status epilepticus management include administering a bolus of an unused “urgent” control medication and then proceeding to pharmacologic coma induction if seizures persist, or moving directly to pharmacologic coma induction [8]. Additional urgent control anti-seizure medications (e.g., phenytoin, valproate, levetiracetam, and phenobarbital) may be reasonable if they have not yet been tried, if the seizures seem to be fragmenting and becoming less frequent, or if the patient needs to be transferred or stabilized prior to administration of continuous infusions. However, preparations should be initiated to achieve definitive seizure control with continuous infusions. Substantial delays have been described before administration of pharmacologic coma induction in children with refractory status epilepticus indicating attention to timing is important [17].

Few data are available regarding management of refractory status epilepticus with midazolam, pentobarbital, and other anesthetic therapies [96]. Midazolam dosing usually involves an initial loading dose of 0.2 mg/kg followed by an infusion at 0.05–2 mg/kg/hour titrated as needed to achieve clinical or electrographic seizure suppression or EEG burst-suppression. Pentobarbital dosing usually involves an initial loading dose of 5–15 mg/kg (followed by another 5–10 mg/kg if needed) followed by an infusion at 0.5–5 mg/kg/hour titrated as needed to achieve seizure suppression or EEG burst-suppression. If seizures persist with midazolam or pentobarbital, then escalating dosing through additional boluses is needed to rapidly increase levels and terminate seizures. Increasing the infusion rate without additional bolus dosing will lead to very slow increase in serum levels, which is inconsistent with the goal of rapid seizure termination. Anesthetics such as isoflurane are also effective in inducing a burst-suppression pattern and terminating seizures but often lead to hypotension, and there are fewer data describing their use. Propofol may rapidly terminate seizures and induce burst-suppression, but it is rarely used in children due to its Federal Drug Administration black box warning due to propofol infusion syndrome.

Patients treated with continuous infusions or inhaled anesthetics require intensive monitoring due to problems including: (1) mechanical ventilation for airway protection and to maintain appropriate oxygenation and ventilation; (2) central venous access and arterial access due to frequent laboratory tests and the possibility of developing hypotension requiring vasopressor or inotropic support; (3) temperature management since high dose sedatives and anesthetics can blunt the shivering response and endogenous thermoregulation; (4) assessment for development of lactic acidosis, anemia, thrombocytopenia, and end organ dysfunction (e.g., acute hepatic or renal injury); and (5) the risk of secondary infections due to indwelling catheters (e.g., central catheters, endotracheal tubes, and foley catheters), as well as some medications (e.g., pentobarbital).

It remains unclear whether the EEG treatment goal should be termination of seizures or induction of burst-suppression. The Neurocritical Care Society’s guideline considers either electrographic seizure cessation of burst-suppression as appropriate goals [8]. It remains unclear how long the patient should be maintained in pharmacologic coma. A survey of experts in status epilepticus management across all age groups reported they would continue pharmacologic coma for 24 h [95]. The Neurocritical Care Society’s guideline recommends 24–48 h of electrographic seizure control prior to slow withdrawal of continuous infusions [8].

Electrographic or electro-clinical seizures frequently recur during weaning of pharmacologic coma medications [97,98,99,100], indicating that pharmacologic coma should be considered as a window during which specific therapies can be instituted for some status epilepticus etiologies and during which other anti-seizure medications can be initiated to provide additional coverage. Only case reports and series are available to guide management at this stage, and the options include topiramate [101,102,103,104,105,106,107], lacosamide [108,109,110], phenobarbital [111,112,113,114], ketamine [115,116,117,118,119], pyridoxine [120,121,122,123,124,125], neurosteroids [126], lidocaine [127,128,129], the ketogenic diet [90,102,130,131,132,133,134,135,136], therapeutic hypothermia [137,138,139,140,141], immunomodulation [142,143], epilepsy surgery [115,144,145,146,147,148,149,150,151,152,153,154], vagal nerve stimulation [155], and electroconvulsive therapy [156,157,158]. These options have been reviewed recently [21,91,94,159].

## 8. Conclusions

Status epilepticus is a common neurologic emergency in children. Management requires simultaneous resuscitation and medical stabilization, diagnosis of the underlying cause, and definitive rapid treatment of both clinical and electrographic seizures. Prompt treatment with benzodiazepines is the first-line treatment of status epilepticus, but many patients will need additional treatment with additional medications including phenytoin, valproic acid, phenobarbital, or levetiracetam. Once an initial benzodiazepine and an additional anti-seizure medication fail to terminate status epilepticus, refractory status epilepticus is diagnosed. Pharmacologic coma induction is needed urgently to terminate status epilepticus, and the period of pharmacologic coma provides an opportunity to further evaluate for precipitants requiring specific management and administration of additional anti-seizure medications.

## Figures and Tables

**Table 1:** International League Against Epilepsy definition of status epilepticus indicates that emergency treatment of status epilepticus should be started at t_1_ and long-term consequences may occur at t_2_. Adopted from Trinka *et al.*, 2015 [6].

Status Epilepticus Type	Time 1 (Treatment Started)	Time 2 (Consequences Expected)
Tonic-clonic	5 min	30 min
Focal with impaired consciousness	10 min	>60 min
Absence	15 min	Unknown

**Table 2:** Dosing recommendations and common side effects for emergent/initial-therapy-phase and urgent/second-therapy-phase anti-seizure medications. Adapted from the Neurocritical Care Society [7] and American Epilepsy Society [8] guidelines for status epilepticus management.

Medication	Recommended Dosing	Serious Adverse Effects	Other Considerations
Emergent/Initial Phase Therapy Phase Medications			
Lorazepam	IV: 0.1 mg/kg IV up to 4 mg per dose, may repeat in 5–10 min	Hypotension Respiratory depression	Dilute 1:1 with saline. IV contains propylene glycol.
Diazepam	IV: 0.15–0.2 mg/kg IV up to 10 mg per dose, may repeat in 5 min Rectal: 0.2–0.5 mg/kg PR up to 20 mg	Hypotension Respiratory depression	Short duration, active metabolite. IV contains propylene glycol.
Midazolam	Adult IM: 0.2 mg/kg up to 10 mg Ped IM: 5 mg if 13–40 kg, 10 mg if >40 kg. 0.3 mg/kg up to 10 mg Intranasal: 0.2 mg/kg Buccal 0.5 mg/kg	Hypotension Respiratory depression	Active metabolite, renal elimination, short duration. For intranasal or buccal, use the IV formulation (5 mg/mL concentration).
Urgent Control Therapy/Second Therapy Phase Medications			
Phenytoin OR Fosphenytoin	20 mg/kg IV, may give additional 5–10 mg/kg 20 mg PE/kg IV, may give additional 5–10 PE/kg	Hypotension Arrhythmias Purple glove syndrome	Phenytoin is only compatible in saline and the IV contains propylene glycol. Fosphenytoin is compatible in saline, dextrose, and lactated ringers solutions.
Levetiracetam	20–60 mg/kg IV	Aggression	Minimal drug interactions, not hepatically metabolized.
Phenobarbital	15–20 mg/kg IV, may give an additional 5–10 mg/kg	Hypotension Respiratory depression	IV contains propylene glycol.
Valproic acid	20–40 mg/kg IV, may give an additional 20 mg/kg	Hyperammonemia Pancreatitis Thrombocytopenia Hepatotoxicity	May be a preferred agent in patients with generalized epilepsy. Avoid if possible hepatic dysfunction, metabolic disease, <2 years old with unknown etiology, pancreatitis, or thrombocytopenia.
